# Optimizing Expectations *via* Mobile Apps: A New Approach for Examining and Enhancing Placebo Effects

**DOI:** 10.3389/fpsyt.2019.00365

**Published:** 2019-05-31

**Authors:** Piotr Gruszka, Christoph Burger, Mark P. Jensen

**Affiliations:** ^1^Mental Health Research and Treatment Center, Faculty of Psychology, Ruhr-Universität Bochum, Bochum, Germany; ^2^Department of Basic Psychological Research and Research Methods, Faculty of Psychology, University of Vienna, Vienna, Austria; ^3^Department of Applied Psychology: Work, Education and Economy, Faculty of Psychology, University of Vienna, Vienna, Austria; ^4^Department of Rehabilitation Medicine, University of Washington, Seattle, WA, United States

**Keywords:** placebo, expectancy, intervention, app, mobile, smartphone, expectation

## Abstract

There is growing interest in interventions that enhance placebo responses in clinical practice, given the possibility that this would lead to better patient health and more effective therapy outcomes. Previous studies suggest that placebo effects can be maximized by optimizing patients’ outcome expectations. However, expectancy interventions are difficult to validate because of methodological challenges, such as reliable blinding of the clinician providing the intervention. Here we propose a novel approach using mobile apps that can provide highly standardized expectancy interventions in a blinded manner, while at the same time assessing data in everyday life using experience sampling methodology (e.g., symptom severity, expectations) and data from smartphone sensors. Methodological advantages include: 1) full standardization; 2) reliable blinding and randomization; 3) disentangling expectation effects from other factors associated with face-to-face interventions; 4) assessing short-term (days), long-term (months), and cumulative effects of expectancy interventions; and 5) investigating possible mechanisms of change. Randomization and expectancy interventions can be realized by the app (e.g., after the clinic/lab visit). As a result, studies can be blinded without the possibility for the clinician to influence study outcomes. Possible app-based expectancy interventions include, for example, verbal suggestions and imagery exercises, although a large number of possible interventions (e.g., hypnosis) could be evaluated using this innovative approach.

## Introduction

There is an increased interest in understanding the effects of placebo interventions and the mechanisms underlying these effects. While basic research has led to a better understanding of psychobiological mechanisms underlying placebo effects by means of strictly controlled experiments (1), applied research has focused on elucidating the factors contributing to placebo effects in clinical practice (2). Some of these studies have been extensively covered in the media, reflecting the interest in placebo effects among the general public. A number of researchers have emphasized the potential of maximizing placebo effects in clinical practice to optimize treatment outcomes (3–5).

Despite recent progress, research in this area faces several unsolved methodological challenges and awaits broader validation. Similarly, as is the case in psychotherapy trials, blinding is extremely difficult to achieve when delivering placebo interventions (6). As a result, it has been challenging to estimate the true effects of placebos separately from the effects of experimenter bias. It is therefore crucial to develop new methods to assess placebo effects.

This paper aims to highlight several methodological advantages of using mobile apps in the area of placebo research. Methodological advantages include full standardization and more reliable blinding, randomization, and allocation concealment. By delivering expectancy interventions *via* apps, researchers can disentangle expectancy effects due to the intervention from effects induced by the patient–researcher (or patient–practitioner) interaction, allowing for the control of experimenter bias (7). Further, combining app-based placebo interventions with experience sampling offers several opportunities for addressing important research questions, such as investigating the impact of placebo interventions on symptom trajectories and on changes in expectations. Additionally, subjective ratings can be potentially complemented by objective data gathered through smartphone sensors and mobile-based experiments. Validated apps can be used for treatment delivery to a large number of people.

### Traditional Definition of Placebo Effects

The term *placebo effect* was first described as a set of positive changes that occur after an inert or inactive treatment (i.e., placebo) was administered to patients (1). Placebo effects are usually associated with so-called blinded randomized controlled trials (RCTs), where placebos—in the form of inert pills, injection, or sham procedures that resemble the active treatments—are administered to study participants in a control group. In order to be considered specifically effective (i.e., beyond the effects of placebos), active treatments are required to outperform placebos in these trials. Optimally, study participants, researchers, study clinicians, data collectors, outcome adjudicators, and data analysts are blinded in RCTs, in order to ensure that differences between active treatments and placebos are not confounded by potential biasing factors such as experimenter effects or participants’ expectations (7–9).

### Problems and Inconsistencies Inherent in the Traditional Definition of Placebo Effects

There are, however, several problems with the abovementioned traditional definition of the placebo effect. First, by defining it as the global response to a placebo treatment, this definition combines the genuine placebo response with other confounding factors, such as natural course or fluctuations in the outcome variable, regression to the mean, the effects of additional treatment(s), observer bias, and subsiding adverse effects of any previous treatments (10, 11). Furthermore, associating placebo effects with RCTs has led to an understanding of placebo effects as (mostly) a vehicle for testing the effectiveness of treatments, such as pharmacological substances, and not otherwise of much interest. As a result, many view placebo effects as something that should be controlled, rather than investigated or used to improve health and function (12).

Second, putting the placebo itself and its inertness into the focus of the definition has led to significant confusion and controversy regarding the placebo effect (e.g., how is it possible for an inert treatment to have genuine effects?). This has resulted in a rather negative connotation being attributed to placebo effects; they are often thought to be fictitious, nonexistent, or only for the gullible [for focus group results, see Ref. (13)]. Furthermore, placebo effects are often considered unworthy and unscientific (14).

As a result of these problems, there have been various attempts to make a case for abandoning the concept of placebo effect (15) and to propose new concepts [e.g., “context effects” (12), “meaning responses” (16)]. Because the concept of placebo is deeply entrenched in the literature, proposed alternative labels and concepts have not been adopted. We have therefore decided to continue using the term *placebo effect* in this paper. However, in order to reconcile this concept with the current evidence, a reconceptualization of this concept is in order [also see Ref. (11)]. In short, the focus should not be on the placebo itself but on the mechanisms underlying the placebo effects. Consistent with this idea, Gliedman and colleagues stated over 60 years ago that the “so-called placebo effect should be looked upon as an epiphenomenon of complicated psychological processes, which are far more important than the disarmingly simple means utilized for its realization” (17).

### Reconceptualization of Placebo Effects

Placebo effects have been found to originate from psychobiological mechanisms in those who respond to placebos (1). Both conscious expectancies and unconscious conditioning mechanisms are assumed to be major contributing factors to placebo responses (1, 18). Previous research has shown that patients’ expectations of clinical benefits play a major role in placebo effects by triggering distinct neurobiological systems that then shape the therapeutic outcomes (3, 11, 19).

When focusing on the underlying mechanism of expectancy learning, it becomes clear that placebo responses are omnipresent in clinical practice—even when no placebo is administered. When active treatments are administered, patients’ responses are determined not only by specific effects of the treatments themselves but also by the patients’ outcome expectations, as well as their possible interaction. This can be easily demonstrated by the so-called “open–hidden” paradigm, which has shown that treatments are more effective when they are given when the patients are present and fully aware of them (i.e., they are able to form expectations) than when they are given in a hidden manner and without patients’ knowledge (11).

A large and growing literature has demonstrated that expectancy-driven placebo effects are a genuine phenomenon that occurs not only after the administration of inert but also of active treatments, and that contributes substantially to the success of many active medical treatments (1). Such effects are potentially relevant in clinical practice because they might lead to better patient health and more effective therapy outcomes. In fact, several studies have shown a positive association between optimistic outcome expectations of patients and favorable therapeutic improvements for a variety of conditions and symptoms, such as disability after surgical interventions (19), hypertension (20, 21), depression (22), anxiety (23, 24), other psychiatric disorders (25), and pain (26).

However, some researchers are less optimistic about the clinical value of placebo effects. Hróbjartsson and Gøtzsche, for example, questioned the clinical relevance of placebos in their meta-analyses (27, 28) and argued that placebos can affect only subjective outcomes such as pain but not objective health parameters. Other researchers, however, note that placebos can improve objective outcomes such as peripheral health parameters and immune responses (29, 30).

### Expectancy Interventions: Modifying Patients’ Expectations to Improve Clinical Outcome

Recently, there has been increased interest in interventions that optimize placebo effects to improve clinical outcomes in routine medical care (1, 2, 31, 32). Previous research has established that interventions targeting outcome expectations have been shown to relieve patients’ symptoms such as pain [for a meta-analysis, see Ref. (26)]. These expectancy interventions usually consist of brief procedures, such as verbal suggestions or imagery interventions, and can be implemented by clinicians in their routine clinical practice. There has been a growing interest in examining the effects of both verbal suggestions and imagery to increase patients’ outcome expectations, which are then thought to enhance treatment outcomes. Such interventions have been used as part of hypnotic treatments for more than a century (33, 34). In fact, evidence indicates that expectancies are mediators of the effects of suggestions both in placebo interventions and in hypnosis (35).

Given that expectancy interventions have been shown to improve symptoms, one could argue that there is an ethical obligation to encourage their widespread implementation and application. This would raise the question regarding how such interventions can be most effectively delivered in order to reach as many patients as possible. Even if the intervention’s benefit is small, it still could be considered a valuable public health intervention if it reaches a high number of people with few adverse effects.

### Methodological Challenges in Validating Clinician-Delivered Expectancy Interventions

Despite the potential of placebo interventions for improving health outcomes, a number of researchers have noted that the efficacy of placebo interventions, such as expectancy interventions, has not been adequately validated. This lack of validation is due to the as-yet-unresolved challenges in placebo research (36, 37), such as the inability to achieve the basic prerequisites for rigorous validity testing of placebo interventions.

One critical precondition is the blinding of the person delivering the interventions. In placebo research—as is also the case in face-to-face psychotherapy trials—reliable blinding of the intervention is extremely challenging. When clinicians are delivering expectancy interventions (e.g., suggesting that pain will decrease soon), they are aware of doing so because delivering the intervention per se is a conscious social act. Thus, they cannot be blinded to treatment allocation or the type of interventions they are delivering. One can envision a variety of ways that this awareness could lead to additional conscious or unconscious changes in the clinicians’ behaviors (e.g., preferential treatment) or verbal/nonverbal communication (e.g., more friendly and reassuring manner) that go beyond the expectancy intervention alone. This lack of blinding may, and probably does, result in experimenter bias (7, 38, 39), which can then contribute to spurious effects or overestimation of effect sizes. Although one might try to blind experimenters or study clinicians by not telling them about study hypotheses, their beliefs and assumptions about the intervention they are delivering can still bias outcomes.

One potential approach to understand the impact of interaction patterns on placebo effects is to manipulate factors within the patient–provider interaction. For example, Kaptchuk and colleagues (40) showed in a single-blind three-arm RCT of 262 patients with irritable bowel syndrome (IBS) that factors such as warmth, empathy, active listening, and indirect suggestions (“I have had much positive experience treating IBS and look forward to demonstrating that acupuncture is a valuable treatment in this trial”) affected outcomes. It makes sense that factors such as clinicians’ warmth, empathy, active listening, or suggestions have positive effects on clinical outcomes, given that similar aspects are at the heart of person-centered psychotherapy (congruence, unconditional positive regard, empathy) and hypnosis (suggestions) (41–46). However, the conclusions that can be drawn from the Kaptchuk et al. (40) and other similar studies are limited because they are generally conducted unblinded. As a result, it is not possible to conclude whether the outcomes are due to these nonspecific clinician factors (e.g., warm, friendly interaction, expectancy manipulation through verbal suggestion) that are a part of how the intervention is delivered, due to experimenter bias (e.g., differential treatment of patients beyond the actual intervention depending on their experimental condition), or both [for a review on the effects of nonverbal behaviors of experimenters on placebo effects in research participants, see Ref. (47)].

Further challenges for the rigorous evaluation of expectancy interventions are response sets, such as acquiescence bias (i.e., the patient or participant wishing to please the experimenter). It is also difficult to disentangle the impact of patient–provider interactions from other response biases. It has been shown, for example, that patients have a higher tendency for response bias when they are experiencing a warm patient–provider interaction (36). Thus, a patient might report a decrease in symptom severity to please the clinician, although it might not reflect an actual change in subjective experience.

In conventional settings, expectancy interventions are delivered by clinicians. These settings almost always involve biases such as those mentioned above. This crucially limits the interpretation of the results. These biasing effects may be an even bigger hindrance for placebo research in children and adolescents, as children are more suggestible than adults (48) and thus might be more easily influenced by experimenter or response bias.

## Advantages of Using Mobile Apps for Placebo Research

There has been an increased interest in apps in the field of medicine and psychology in recent years. Mobile apps are being used more and more frequently by researchers, clinicians, and patients and have the potential to revolutionize different aspects of medical and psychotherapeutic care (49–54). However, to the best of our knowledge, apps have not been systematically used to examine or deliver placebo-boosting interventions. Thus, the field could potentially profit from technological advances in the area of smartphones.

**Figure 1:**
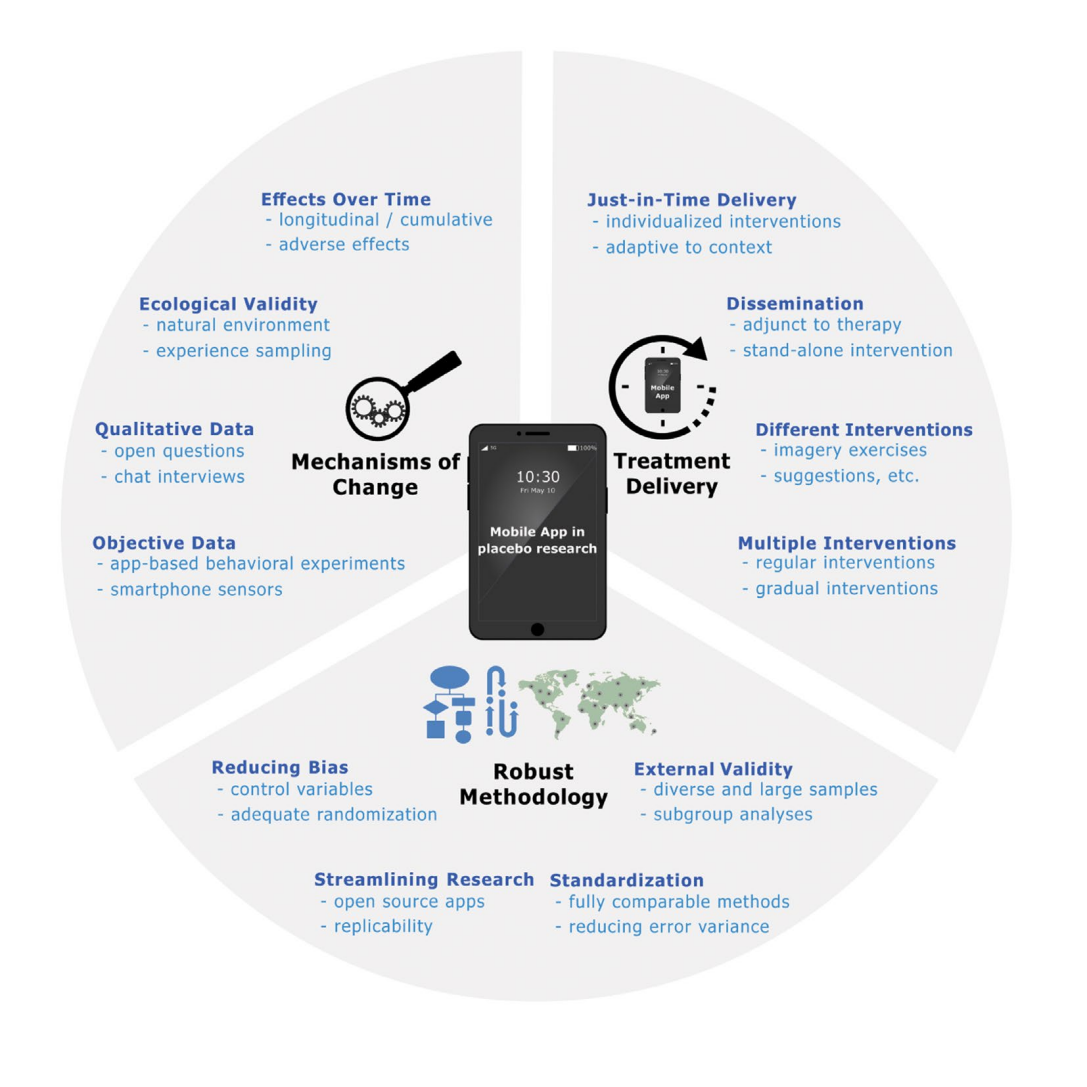
Schematic overview of advantages of app-based expectancy interventions.

We propose in this paper that the use of mobile apps can lead to many advantageous developments in both placebo research and clinical practice: i) using smartphones can help to solve problems inherent in validating placebo-boosting interventions such as expectancy manipulations; ii) mobile apps can be used to gain a better understanding of placebo mechanisms in everyday life; and iii) once placebo-boosting interventions have been successfully validated, apps can be used as an effective way to deliver these interventions as an adjunct to therapy sessions or as a stand-alone tool to a large number of people (see **Figure 1**).

### Validating Placebo-Boosting Interventions

In light of difficulties in reproducing major findings in psychological and medical science (55–58) in recent years, the area of placebo research might profit from innovative methodological advances. App-based studies offer several methodological advantages enabling more robust research, which can play an important role in improving the scientific status of expectancy interventions, potentially enabling them to be introduced into mainstream medicine (1).

One of the advantages of app-based expectancy interventions relates to the fact that they can be fully standardized. In the past, expectancy manipulations used different protocols and were conducted in different settings and with different samples. Thus, differences in outcomes may be related not only to different outcome measures and types of illnesses (29, 59) but also to different protocols, settings, clinicians, samples, sampling procedures, and methodological standards.

To address these issues, app-based interventions can take advantage of full standardization, thereby reducing heterogeneity. They can also ensure adherence to key characteristics of high-quality trials such as adequate randomization and allocation concealment, by performing these tasks objectively and reliably within the app. This can be achieved by placing importance on the right timing; that is, randomization and allocation concealment can be performed by the app after the clinic or lab visit (where the patient or study participant can be introduced to the app) so that it is impossible for inadequate group allocation or blinding to impact the experimenter or clinician and his or her interaction with the study participant. Alternatively, patients might use the app fully remotely without any contact at all with experimenters or clinicians. As it has been shown that trials with inadequate or unclear allocation concealment exaggerate subjective outcome effects (8, 60), the use of apps could potentially increase the reliability of effect size estimates.

By making the apps open-source, independent researchers could use them at minimal cost to conduct fully identical replications. Although innovative placebo interventions [e.g., open-label placebos (61, 62)] have been tested in recent years, identical replications of these studies are lacking. Given that effect sizes in psychology are, on average, only half the initial size when replicated (56), identical replications are crucial for validating expectancy interventions. Providing app-based expectancy interventions as open-source software may potentially reduce costs by streamlining research (63), thereby increasing the quality of conducted studies (64).

The standardization that results from the use of app-based expectancy interventions would lead to smaller heterogeneity and more precise replications. Thus, studies will be fully comparable and could be easily aggregated in prospective meta-analyses (65), leading to large and meaningful sample sizes, a key characteristic of robust research (66, 67). This would also allow investigators to quantify the influence of sample procedures and sample characteristics on trial outcomes and replication rates.

In addition, by standardizing expectancy interventions and increasing sample sizes, variance will be reduced. This could enable researchers to investigate the impact of expectancy interventions in different samples. Thus, conducting highly standardized app-based experimental interventions in different samples and cultures can lead to a better understanding of interpersonal and intercultural differences in expectations (68). This might lead to more precise predictions of placebo effects and to the development of more effective culturally sensitive expectancy interventions. As most research is conducted in Western, educated, industrialized, rich, and democratic (WEIRD) (69) samples, app-based expectancy interventions offer the potential of gathering data in more diverse and representative samples if uploaded to an app store or used as an add-on to established treatments.

Crucially, experimenter bias can be limited by disentangling patients’ expectations from the patient–provider interaction. By delivering expectancy interventions within the app, expectations can be studied in isolation, disentangled from the effects of the patient–provider interaction. As such, expectancy interventions can be delivered at home, after seeing the clinician, thereby eliminating experimenter biases (7, 39), or even fully remotely if the app is uploaded to an app store.

### Gaining Insights Into Placebo Mechanisms in Everyday Life

#### Ecological Validity

Further, app-based expectancy interventions offer the potential to deliver interventions with high ecological validity in patients’ everyday life. Thus, effects from the lab can be extended to the natural surroundings of patients, thereby increasing the potential usefulness of interventions (64). Apps also offer the opportunity of combining experience sampling procedures with expectancy interventions. Although the advantages of experience sampling methodology have been discussed in the area of psychiatry before [e.g., Ref. (70)], to our knowledge, this methodology has not yet been applied to placebo research. Experience sampling is a method for assessing momentary thoughts, feelings, and symptoms and is usually employed several times per day over consecutive days (71, 72). This structured diary method can be easily implemented in mobile apps. It offers the possibility to assess symptom trajectories in everyday life as well as underlying mechanisms, thereby increasing ecological validity.

Investigating symptom trajectories over time could enable researchers to cluster study participants into different types of responders (73). Gueorguieva and colleagues (74), for example, have investigated trajectories of depression severity in clinical trials of duloxetine showing that placebo-treated patients were characterized by different trajectories than responders and nonresponders in the antidepressant-treated subsample. Moreover, it may be possible to differentiate study participants based on early or late responses. Simons and colleagues (75) have classified response trajectories of children with chronic pain after intensive pain rehabilitation treatment into early treatment responders, late treatment responders, and nonresponders.

In addition, more intensive daily experience sampling would enable researchers to investigate the variability in symptoms within and between persons following expectancy interventions. Apps might potentially enable researchers also to gather information on adverse events and long-term data after expectancy interventions. Thereby it would be possible to answer an important research question that has not yet been adequately addressed: Do expectancy interventions lead to long-lasting changes or only temporary improvement? Thus, this type of research has the potential to elucidate a much more in-depth understanding of placebo effects in everyday life.

Experience sampling might be used to assess not only symptom fluctuations but also changes in symptom expectations. Mun and colleagues (76) have, for example, investigated pain expectations in a sample of 231 individuals with rheumatoid arthritis showing that pain expectations are a reliable predictor of pain. As expectations are at the heart of placebo effects, the assessment and fluctuation of symptom expectations will add to a more precise understanding of placebo effects and a better understanding of expectations and their formation over time.

The assessment of symptoms and expectations *via* apps can be complemented by open questions and other qualitative assessments (e.g., interviews *via* smartphone chats about daily experiences) to investigate the impact of not only expectancy interventions but also daily experiences such as social interactions on symptom trajectories and expectations. A detailed understanding of these processes will enable a more precise prediction of placebo effects and will offer new avenues for individualized expectancy interventions.

#### Assessment of Objective Data

Subjective data on symptom and expectation trajectories can be complemented with data obtained through smartphone sensors. Smartphone sensors can provide researchers with data about social interactions, daily activities (e.g., physical activity and sleep quality), and mobility patterns (77, 78). Researchers targeting chronic pain could, for example, investigate how expectancy interventions affect physical activity, sleep quality, or social interactions.

Apps also offer the possibility of running behavioral experiments on smartphones. Thus, experiments from the lab could be conducted on smartphones. Free popular experimental software such as PsychoPy^1^ is now also available for mobile devices (79, 80), potentially enabling researchers to conduct these experiments with minimal costs. A promising approach might be to develop experiments to phenotype beliefs underlying changes in expectations or to employ existing implicit measures such as the implicit association test (IAT) (81) for that purpose.

Although some researchers have argued that placebo effects lead only to an improvement in parameters that depend on subjective patient ratings (28), others came to more favorable conclusions (29). Thus, it seems crucial to find alternative ways of assessing objective data following expectancy interventions in order to resolve this issue. Smartphones and other mobile devices offer several efficient ways for doing so by assessing different types of behavioral measures in an unobtrusive way without putting additional burden on study participants.

### Treatment Delivery

#### Multiple or Repeated Interventions

Apps also can be used to deliver multiple or repeated expectancy interventions, thereby potentially increasing their efficacy. One could, for example, deliver different weekly expectancy interventions and assess their impact on symptom trajectories through the use of experience sampling. This might potentially enable researchers to investigate cumulative effects of repeated expectancy interventions. As some patients show *cognitive immunization strategies* [strategies to weaken or eliminate expectation violation or, in other words, strategies to reduce cognitive dissonance between suggested information and individual beliefs, (82, 83)], it might be necessary to deliver expectancy interventions gradually or to individualize them according to patient beliefs, person characteristics, and symptom trajectories for them to take effect.

#### Just-in-Time Adaptive Expectancy Interventions

A precise understanding of symptom and expectation trajectories complemented with behavioral data through smartphone sensors might pave the way for the development of just-in-time adaptive expectancy interventions (JITAEIs). Just-in-time adaptive interventions (JITAIs) relate to interventions that are adapted to the status or context of an individual over time (84–87). As every person has individual beliefs, it is likely that individualized interventions will have higher efficacy. Psychotherapy research has shown, for example, that resistant patients profit more from nondirective therapy than from directive approaches (88). Thus, patients with more rigid health beliefs, which make them more resistant to change, could potentially profit more from indirect suggestions (“Many patients profited from the app before”) or imagery exercises (e.g., imagining healthy future self) than from direct suggestions (“You will profit from this app”). Suggestions as part of expectancy interventions might therefore be delivered based on symptom changes, patients’ beliefs and needs, other personal characteristics, and data from smartphone sensors. Thus, if patients have strong beliefs about their condition (as assessed by questionnaires) and have not shown symptom improvements for several weeks, they might be offered indirect suggestions, such as, “Some patients did not seem to profit from the app in the beginning, some were even frustrated. Often, however, their symptoms did in fact improve, bit by bit.” Less resistant patients, who report early improvements in symptom reduction, might be given more direct suggestions such as, “You have used the app for one week now. Your pain has already decreased. You will experience your pain decreasing even further in the coming weeks.” Thus, the app may be programmed in such a way as to accommodate the patients’ symptom ratings, other personal characteristics, and objective data gathered through smartphone sensors to deliver individualized expectancy interventions.

#### Treatment Dissemination

Once an expectancy intervention is found to be effective for producing changes in reported symptoms, clinical implementation of that intervention may prove challenging, given the significant limitations on clinicians’ time. Apps could potentially be used to deliver highly standardized expectancy interventions without posing an unnecessary burden on busy clinicians. Thus, app-based expectancy interventions might be used either as an add-on to existing medical and psychotherapeutic procedures or even as a stand-alone intervention.

## Discussion

The present paper introduces a novel approach of delivering expectancy interventions (e.g., verbal suggestions, imagery exercises) aimed at boosting placebo effects through mobile apps. Because this approach does not involve an attendant person (e.g., an experimenter or clinician) to deliver the expectancy intervention, expectancy-driven components of the intervention can be disentangled from social interaction–driven components. Such an approach can answer questions such as what aspect of the placebo effect is driven by changes in expectancies. Moreover, this approach can help us to better understand the patient populations for whom such interventions may be most effective.

Previous studies have already shown that verbal suggestions delivered by technology (i.e., audio players) are effective in improving clinical symptoms in patients. For example, playing recorded hypnosis audio tracks, consisting of verbal suggestions (also used to elicit imagery), has been shown to be effective in reducing pain [e.g., Refs. (89, 90)]. These studies, however, did not use mobile apps to deliver the verbal suggestions and thus did not exploit the full potential of available technology. Nevertheless, the findings support the approach presented in this paper as promising.

As expectancy interventions have been used primarily in experimental research in relation to an active or placebo treatment [oral, injection, cutaneous, or other; see Ref. (26)], their implementation in clinical settings may be inspired by clinical hypnosis research. Clinical hypnosis has a long history of using verbal suggestions for symptom improvement (33, 34), with several journals focusing solely on hypnosis (e.g., *American Journal of Clinical Hypnosis*, *International Journal of Clinical and Experimental Hypnosis*). Bringing both fields together—research on clinical hypnosis and placebo research—may be particularly fruitful for developing more effective expectancy interventions. Thus, we think that new expectancy interventions for mobile apps would greatly profit from research in both the clinical hypnosis and placebo research fields. However, possible app-based expectancy interventions are not limited to verbal suggestions and imagery exercises, as a large number of expectancy interventions could be delivered and evaluated using this innovative approach.

We have described several advantages of app-based expectancy interventions (see **Table 1** for an overview). This approach makes it possible to investigate placebo effects independent of the patient–provider interaction, thereby overcoming some of the inherent methodological challenges associated with placebo interventions. Highly standardized app-based expectancy interventions can lead to more robust research by enabling researchers to replicate findings more easily. Apps also can be used to phenotype placebo responses longitudinally, while investigating mechanisms of change. Researchers can integrate behavioral experiments into their apps and gather data from smartphone sensors for this purpose. This could allow researchers to predict placebo responses more precisely, helping scientists gain insights into short-term, long-term, and cumulative effects of expectancy interventions as well as adverse events. Further, app-based expectancy interventions can be individualized and delivered just in time.

**Table 1:** Summarized advantages of app-based expectancy interventions.

Validating placebo-boosting interventions	
Full standardization	Fully standardized placebo interventions are fully comparable, result in smaller heterogeneity, and can be easily aggregated, leading to large and meaningful sample sizes; this will enable investigating predictors of placebo responses in subgroups of patients.
Adequate randomization and blinding	Randomization can be conducted within the app, thereby ensuring adequate randomization and allocation concealment. Interventions can be delivered in the absence of the clinician, thereby ensuring reliable blinding.
Open-source apps	Releasing app-based expectancy interventions as open-source might enable other research groups and clinicians to conduct similar studies with little costs, thereby enabling easy-to-implement replications.
More diverse samples	Apps enable conducting expectancy interventions in more diverse samples and different cultures.
Limiting experimenter bias	Expectations can be studied in isolation from the effects of the patient–provider interaction, allowing disentangling patient expectations from effects of the patient–provider interaction.
Gaining insights into placebo mechanisms in everyday life	
Ecological validity	Symptom and expectation trajectories can be studied in everyday life, thereby increasing ecological validity and enabling individualized expectancy interventions.
Adverse events	Questions about adverse events can be easily integrated into apps, thus allowing gathering data on potential short- and longer-term adverse events due to expectancy interventions.
Long-term and cumulative effects	Long-term and cumulative effects of expectancy interventions can be assessed *via* experience sampling.
Qualitative data	Apps can be used to gather qualitative data (open questions, chat interviews) on the impact of expectancy interventions to understand the formation of expectations.
Objective data	Subjective patient ratings can be complemented with objective data by using behavioral experiments on smartphones and gathering data from smartphone sensors.
Treatment delivery	
Multiple interventions	The effectiveness of app-based expectancy interventions can be increased by delivering them multiple times.
Just-in-time adaptive expectancy interventions	App-based expectancy interventions can be individualized and delivered just in time to fit individual beliefs, personal characteristics, symptom trajectories, and objective data.
Treatment dissemination	App-based expectancy interventions can be uploaded to app stores and delivered as an add-on to existing medical and psychotherapeutic procedures or as a stand-alone intervention.

This approach has significant potential for both research and clinical practice. If simple app-based interventions aimed at improving outcome expectations (e.g., verbal suggestions or imagery exercises) lead to symptom relief, they could be widely applied to optimize patient treatment. Such approaches could be used to support medical treatment more efficiently (e.g., reducing dose of medication without diminishing effects, improving outcome effects without having to raise medication dose) or even be a viable alternative to medication when the anticipated adverse effects might outweigh the benefits of drug use (91). In the field of pediatrics, where medications may have long-term side effects on children’s brain development, reducing the pharmacological load might be even more relevant. Improving outcome expectations could also translate into better patient adherence and compliance (32) and reduced feelings of helplessness and hopelessness.

However, since no effectiveness data on different forms of app-based expectancy interventions are currently available, it will take further empirical research efforts to understand the kinds of expectancy interventions that are most effective under what conditions and for what populations. Eventually, it will be necessary to conduct studies with large samples to investigate precise predictors of placebo responses taking into account various data sources, including data from smartphone sensors, app-based experiments, as well as biological data. These studies will provide important information for individualizing interventions, which could subsequently be delivered just in time.

Some limitations of this approach need to be acknowledged. The first limitation refers to the fact that mobile apps cannot replace the provider–patient relationship, which is considered an important factor of placebo effects and clinical outcomes. Rather, their strength lies in their ability to systematically study expectation effects separately from social interaction effects. App-based approaches might be of special interest for i) patients who do not want to disclose their problems to clinicians and ii) patients with a high affinity for smartphones and new technology, such as children and adolescents (92). They may also be used as an add-on or aftercare to medical/psychotherapeutic procedures.

The second limitation refers to legal, ethical, and privacy-related aspects of app-based treatments. Apps that aim to treat medical conditions are considered medical devices and need to adhere to relevant regulations, such as the European Medical Device Regulation or the Food and Drug Administration (FDA) regulation in the US before entering the market (93, 94). Furthermore, using medical apps in research potentially leads to challenges relating to consent and privacy (47). The fact that the legal situations regarding medical privacy vary between countries further complicates the matter (95). Several recommendations have been made to address ethical issues, data privacy, and data security concerns, which should be considered while developing mobile apps (96–99).

The introduction of app-based interventions also comes with technological challenges. First, although there are currently only two major operating systems available for smartphones (Android and iOS), new versions of these operating systems are released continuously. Most manufacturers also provide modified versions of Android, resulting in potential compatibility issues. In addition, manufacturers provide smartphones and other mobile devices with a plethora of different hardware specifications, including different screen sizes and screen resolutions. Thus, software developers not only need to ensure that the apps run on different operating systems but also need to program them with different screen sizes, screen resolutions, and hardware specifications in mind.

Second, it has been proposed that interventions that are delivered through a mobile device might lead to heightened expectations of a high-tech treatment among patients with high affinity for their digital devices. This phenomenon has been termed “digital placebo” (100). It has been argued that trials with such app-delivered interventions have to be complemented with an active placebo control group that also involves an app (101, 102) in order to distinguish the specific effects of the app-delivered interventions from digital placebo effects.

Further, the use of mobile technology and the Internet might be contraindicated for individuals prone to Internet addiction (103). These individuals might not profit from such apps and therefore should be assigned to other treatment modalities. Also, different operating systems or smartphone technologies in general might represent confounders that could bias the results. For example, the majority of the population in Europe uses Android smartphones, whereas there is a higher proportion of iPhone users in the US. There might also be sociodemographic differences between Android and iOS users (104).

Finally, we want to point out the importance of future research efforts to focus on translational aspects of their findings. It is well established that many findings from studies evaluating the efficacy of behavioral and health promotion interventions have not been put into (clinical) practice. It has been pointed out that an important reason for this gap between research results and evidence-based practice may lie in the tendency of the current research culture to neglect issues of external validity (105, 106). To address this important issue, Glasgow and colleagues argue that researchers should pay attention to issues of moderating variables (external validity) in both efficacy and effectiveness studies (107). These issues also have been shown to be present in smartphone-enhanced health research, as mobile health intervention studies tend to neglect the reporting of validity indicators, including indicators of external validity (108). Although there may be practical constraints, the usefulness of future research efforts (64) might benefit from quality criteria available from published best practice standards [e.g., Consolidated Standards of Reporting Trials of Electronic and Mobile HEalth Applications and onLine TeleHealth, CONSORT-EHEALTH (109)] and evaluation frameworks [e.g., RE-AIM framework: reach, efficacy/effectiveness, adoption, implementation, maintenance (105, 110)]. These criteria might be used at different stages throughout the research process (reviewing of literature, planning, conducting, reporting) as a guide to maximize internal and external validity. These criteria include, among others, reports on sample representativeness, research setting and delivery agents, theoretical framework, the development process, source code, accessibility and features/functionalities of the app, information on instructions/reminders/prompts, sustainability of effects, and potential conflicts of interest.

Once the above-described issues have been adequately tackled and the external validity of apps addressed, the use of apps and big data could potentially open up completely new avenues of research and contribute to truly personalized and more effective treatments. We have only touched upon some of the possibilities of smartphone technology in the area of placebo research. There will be many more approaches to come in the future, which we cannot even imagine right now.

## Author Contributions

PG, CB, and MJ conceived and designed the paper. PG and CB wrote the first draft of the manuscript. PG, CB, and MJ wrote the final version.

## Funding

The authors acknowledge support by the DFG Open Access Publication Funds of the Ruhr-Universität Bochum.

## Conflict of Interest Statement

The authors declare that the research was conducted in the absence of any commercial or financial relationships that could be construed as a potential conflict of interest.

## Footnotes

1
https://www.psychopy.org/
